# The luminance ratio of autofluorescence in a xenograft mouse model is stable through tumor growth stages

**DOI:** 10.1002/cre2.126

**Published:** 2018-08-15

**Authors:** Shigeki Sumi, Naoki Umemura, Makoto Adachi, Takahisa Ohta, Kosuke Naganawa, Harumi Kawaki, Eiji Takayama, Nobuo Kondoh, Shinichiro Sumitomo

**Affiliations:** ^1^ Department of Oral and Maxillofacial Surgery Asahi University School of Dentistry Japan; ^2^ Department of Oral Biochemistry Asahi University School of Dentistry Japan

**Keywords:** autofluorescence imaging, biochemical tumor markers, diagnostic equipment, flavin adenine dinucleotide, oral cancer

## Abstract

The aim of this research was to investigate the value of autofluorescence imaging of oral cancer across different stages of tumor growth, to assist in detecting tumors. A xenograft mouse model was created with human oral squamous cell carcinoma cell line HSC‐3 being subcutaneously inoculated into nude mice. Tumor imaging was performed with an autofluorescence imaging method (Illumiscan®) using the luminance ratio, which was defined as the luminance of the tumor site over the luminance of normal skin tissue normalized to a value of 1.0. This luminance ratio was continuously observed postinoculation. Tumor and normal skin tissues were harvested, and differences in the concentrations of flavin adenine dinucleotide and nicotinamide adenine dinucleotide were examined. The luminance ratio of the tumor sites was 0.85 ± 0.05, and there was no significant change in the ratio over time, even if the tumor proliferated and expanded. Furthermore, flavin adenine dinucleotide and nicotinamide adenine dinucleotide were significantly lower in tumor tissue than in normal skin tissue. A luminance ratio under 0.90 indicates a high possibility of tumor, irrespective of the tumor growth stage. However, this cutoff value was determined using a xenograft mouse model and therefore requires further validation before being used in clinical diagnosis.

## 1 INTRODUCTION

Oral squamous cell carcinoma (OSCC) represents more than 95% of all malignant neoplasia of the oral cavity (Muir & Weiland, 1995). For OSCC diagnosed in the early phase (stages I–II), the 5‐year survival rate is approximately 80% when effective treatment is performed, and early detection is thought to affect the prognosis (Ferreri et al., 2010; Petersen, 2009; Scott, Grunfeld, & McGurk, 2005; Shin, Vigneswaran, Gillenwater, & Richards‐Kortum, 2010; Vernham & Crowther, 1994). In many cases, patients who are aware of discomfort in the oral cavity will visit a general dentist. Therefore, general dentists have an important role to play in the early detection of oral mucosal lesions (Pentenero, Marino, Tempia Valenta, Navone, & Gandolfo, 2014; Pentenero, Val, Rosso, & Gandolfo, 2018).

**Figure 1:**
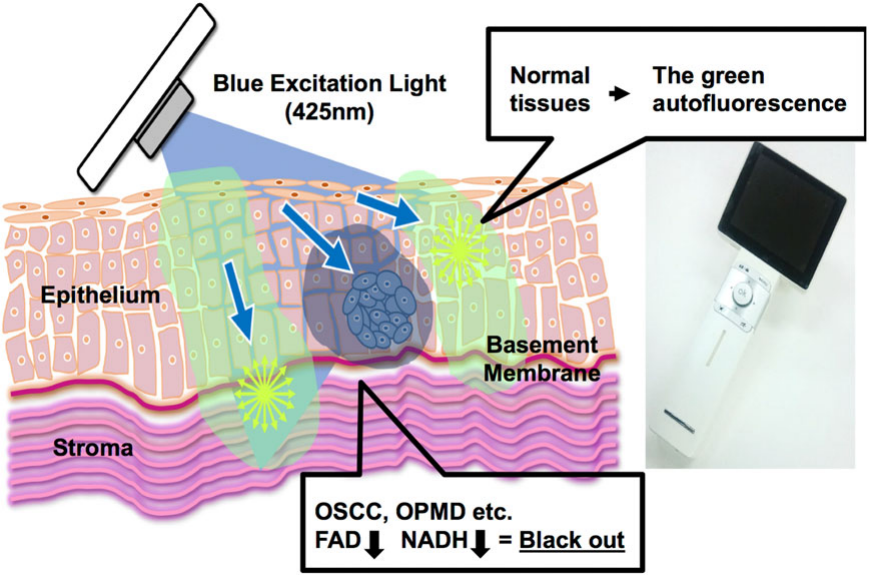
Mechanism of the autofluorescence imaging device. Blue excitation light from the autofluorescence visualization device (Illumiscan®) is emitted onto the oral mucosa, and endogenous autofluorescent substances (FAD and NADH) in the healthy normal tissues emit green light. FAD and NADH levels decrease in OSCC and OPMD, and therefore, OSCC and OPMD can be detected as fluorescence visualization loss, which is shown as a darkened region. FAD: flavin adenine dinucleotide; NADH: nicotinamide adenine dinucleotide; OPMD: oral potentially malignant disorders; OSCC: oral squamous cell carcinoma

Currently, the differentiation of oral potentially malignant disorders (OPMD; including oral leucoplakia, erythroplakia, proliferative verrucous leukoplakia, Viadent‐related leukoplakia, verrucous hyperplasia, keratoacanthoma, and oral submucous fibrosis) from OSCC requires medical diagnoses such as a medical interview, exfoliative cytology, and toluidine blue staining, with the ultimate final pathological diagnoses being performed in medical institutions such as university hospitals (Luo et al., 2016; Mortazavi, Baharvand, & Mehdipour, 2014; Rhodus, 2009). When OSCC is suspected, incisional biopsy is usually performed; this requires excision of the lesion and part of the healthy tissue (Pentenero et al., 2014; Pentenero et al., 2018). In contrast, oral examination at a general dental practice is mainly based on inquiries made at the side of the dental chair and palpation, and judgments are often based on the experience and knowledge of the individual dentist (Messadi, 2013). Therefore, a technically simple OSCC screening method that could be quickly performed with reduced patient burden is strongly desired (Osman, Costea, & Johannessen, 2012). In recent years, one such possible diagnosis method involving the application of autofluorescence imaging has been increasingly used. Currently, the VELscope® (LED Dental Inc., Vancouver, Canada; Awan, Morgan, & Warnakulasuriya, 2011; Ganga et al., 2017; Hanken et al., 2013; Huang et al., 2017; Yamamoto et al., 2017) and DIFOTI® (Electro‐Optical Sciences Inc., Irvington, NY, USA; Bin‐Shuwaish, Yaman, Dennison, & Neiva, 2008) systems have been launched for general dental clinics (Lingen, Kalmar, Karrison, & Speight, 2008). These instruments can detect a decrease or increase in the autofluorescence of flavin adenine dinucleotide (FAD), nicotinamide adenine dinucleotide (NADH), or collagen cross linking, by irradiating the lesional tissue with blue light of a wavelength of 400 to 460 nm (Laronde et al., 2014; Lingen et al., 2008). The Illumiscan® system, which was launched by Shofu Inc. (Kyoto, Japan) in 2015, is similar to the VELscope®, in that it irradiates a lesion site with blue excitation light of 425‐nm wavelength. This allows observation of the fluorescence visual loss (FVL), which thereby reflects decreases in fluorescent substances such as FAD or absorption of light by hemoglobin in the tumor tissue (Figure 1). Normal tissue appears green on imaging, because of the fluorescence stimulated by the blue excitation light. Conversely, areas showing disease involvement appear darkened on the images. The difference between the Illumiscan and conventional devices is that the Illumiscan is an all‐in‐one device and includes the excitation‐light emitting part and a monitor, with the handle being equipped with a focus adjustment function; moreover, this device is specialized for the detection of fluorescence from FAD, as it has a fluorescence filter that interrupts wavelengths shorter than 470 nm (Figure 1). Since 2015, our department has been performing experimental trials on this autofluorescence imaging device (Illumiscan®) in patients suspected of OSCC or OPMD in the outpatient clinic.

Over the past three decades, the diagnostic accuracy of autofluorescence has been evaluated worldwide, not only for OSCC, but also for lung cancer, esophageal cancer, gastric cancer, colorectal cancer, and dysplastic or neoplastic lesions (Divisi, Di Tommaso, De Vico, & Crisci, 2010; Kato, Kaise, Yonezawa, Yoshida, & Tajiri, 2007; Mayinger et al., 2001). Lane et al. (2006) reported relatively high screening accuracy for autofluorescence in the oral cavity. In contrast, Farah, McIntosh, Georgiou, and McCullough (2012) reported that the sensitivity of VELscope was low and that it cannot be used as a definitive diagnosis for epithelial dysplasia. Therefore, further discussion and research is necessary to improve screening accuracy with such methods.

A number of studies on the autofluorescence imaging method have been concerned with validating the differential diagnosis of OSCC and OPMD, by making comparisons between the FVL of the suspected lesion and cytodiagnosis or biopsy (Awan et al., 2011; Elvers et al., 2015; Ganga et al., 2017; Petruzzi et al., 2014; Scheer et al., 2016; Yamamoto et al., 2017). However, there is currently no standardized numerical cutoff value for assisting in the identification of tumors using such autofluorescence visualization devices. Furthermore, the relationship between FVL and tumor growth is unclear. Therefore, although we have started to introduce the use of autofluorescence visualization into the outpatient clinic to examine patients suspected of OSCC, it is difficult to judge whether a lesion is a tumor or not according to only the autofluorescence imaging, even if FVL is observed.

In this study, we aimed to demonstrate a cutoff value for differential diagnosis of OSCC by observing changes in luminescence and luminescence ratios over time in an OSCC xenograft mouse model. Furthermore, we also used molecular biology techniques to examine factors causing the FVL in the autofluorescence imaging.

## 2 MATERIALS AND METHODS

### 2.1 Mice

Six‐week‐old male BALB/c nude mice were obtained from Chubu Kagaku Shizai Co., Ltd. (Nagoya, Japan). The mice were maintained in an animal rearing hood (Chiyoda TechnoAce Co., Ltd., Nagoya, Japan) under constant conditions of humidity 60% ± 10 and air temperature 22 ± 3. This study was approved by the Animal Ethics Committee of Asahi University (No. 16‐042 and 17‐033).

### 2.2 Cell culture

The HSC‐3 human OSCC cell line was obtained from Riken Cell Bank (Ibaraki, Japan). Cells were cultured in Dulbecco's modified Eagle's medium (Life Technologies Japan Ltd.) supplemented with 10% FBS (Life Technologies Japan Ltd.) and antibiotics (penicillin, 100 U/ml; streptomycin, 100 μg/ml; amphotericin B, 25 μg/ml) at 37 °C in a humidified atmosphere containing 5% CO2.

### 2.3 Xenograft mouse model

Approximately 2 × 10^6^ HSC‐3 cells were suspended in 0.1 ml of phosphate‐buffered saline and were subcutaneously inoculated into the lateroabdominal area of the mice. Tumor size was observed every week, with the tumor volume being calculated using the formula a × b^2^/2, where “a” is the length and “b” is the width of the tumor diameters. In the same observation periods, the tumors were photographed with ordinary and autofluorescence imaging using the Illumiscan system. The procedures were repeated on a total of 10 mice to generate a tumor growth curve. At the end of this experiment, the mice were given 5% isoflurane inhalation anesthesia and were euthanized by an intraperitoneal pentobarbital Na overdose (approximately 100 mg/kg).

### 2.4 Analysis of autofluorescence imaging

**Figure 2:**
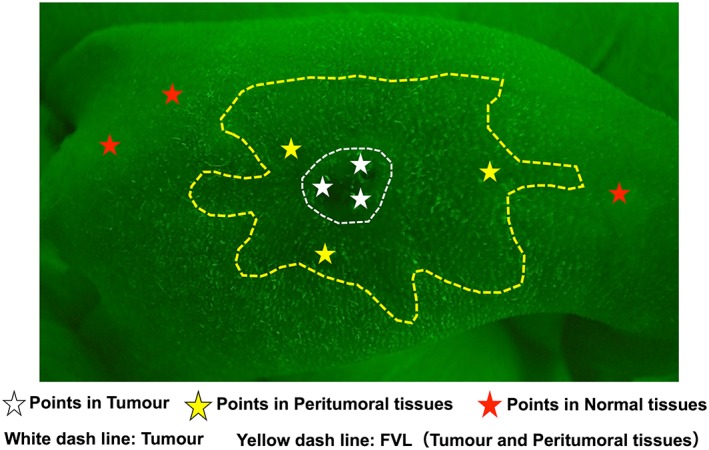
Representative autofluorescence image in a xenograft mouse model. The white dashed line circumscribes a tumor, the yellow dashed line shows a region with fluorescence visual loss (FVL; tumor and peritumoral tissues), and the other areas are normal tissues. Three points were selected from each region, and the mean brightness of these points was measured using the image analysis software

The Illumiscan® autofluorescence system was launched by Shofu Inc. (Kyoto, Japan) in 2015, with the intended use being mainly in Japanese dental practices. The autofluorescence imaging obtained by the Illumiscan was transferred to a personal computer and image analysis was performed with the Illumiscan image analysis software. This software was developed for research purposes only. Images were divided into three fields, the tumor parenchyma, its surrounding tissue, and normal mouse skin tissue. In the xenograft mouse model, tumor masses were palpable from 7 days after subcutaneous transplantation into the mouse. Therefore, we defined the tumor mass site as the tumor parenchyma, the surrounding area as the surrounding tissue, and the area outside of this as the normal mouse skin tissue. Three points were selected from each of the fields, and the luminance values at these points were quantified (Figure 2) and averaged to form the luminance value of the field. The luminance values of tumor parenchyma and surrounding tissue were then compared with the luminance of normal tissue, and a luminance ratio (luminance ratio = the luminance of the lesion/the luminance of normal tissue) was calculated with the luminance value of the normal tissue being normalized to a value of 1.0. In addition to the autofluorescence imaging, images of the tumor masses and surroundings were taken with a normal camera.

### 2.5 FAD assay

On Day 35 after tumor inoculation, the tumor tissue and normal mouse skin tissue were harvested from the mice. The skin of the mouse was opened and turned over, and the tumor mass was peeled from the mouse skin as a single mass. The tissues were floated in 400 μl of FAD buffer attached to a FAD assay kit (Abcam Technology: ab204710, Cambridge, UK) and mashed on ice using a BioMasher II 1.5‐ml tube‐size disposable homogenizer (Nippi Incorporated., Tokyo, Japan). The quantity of protein was then determined using a Pierce™ 660‐nm protein assay reagent (Thermo Fisher Scientific), and each sample was diluted to 50‐μg protein. A deproteinization step was then performed using a deproteinizing sample preparation kit‐TCA (Abcam: ab204708), and a fluorometric assay was undertaken with measurement at Ex/Em = 535/587 nm with a TECAN SpectraFluor plus and XFluor4 software (Tecan Japan Co., Ltd., Kawasaki, Japan).

### 2.6 NAD/NADH assay

For the NAD/NADH assay, tissues were floated in 400 μl of NADH/NAD extraction buffer attached to an NAD/NADH assay kit (Abcam: ab65348) and were then mashed on ice using a BioMasher II 1.5‐ml tube‐size disposable homogenizer. The quantity of protein was then determined using a Pierce™ 660‐nm protein assay reagent (Thermo Fisher Scientific), and each sample was diluted to 50‐μg protein. For the deproteinization step, samples were filtered through a 10‐kD spin column (Abcam: ab93349). The samples were then divided into two, with one unprocessed sample being treated as NADt (total NAD and NADH), and the other being further processed according to the instructions to prepare NADH (NAD decomposed sample). The absorbance was measured at Optical density (OD) 450 nm with a TECAN SpectraFluor plus and XFluor4 software.

### 2.7 Statistical analysis

Differences in luminance values between tumor site, peritumoral tissues, and normal tissue, and differences in NADt and NADH values between normal tissue and tumor tissue, were evaluated using one‐way analysis of variance followed by the Tukey–Kramer test. Differences in measured FAD values between normal tissue and tumor tissue were evaluated using an unpaired two‐tailed student's *t* test. Differences between tumor/normal tissue luminance ratios and peritumoral/normal tissue luminance ratios, irrespective of the tumor growth stage, were evaluated with the Tukey–Kramer test. A value of *p* < 0.05 was considered statistically significant. All statistical analyses were performed using Statistics Analysis software for Mac Ver. 3.0 (ESUMI Co. Ltd., Tokyo, Japan).

## 3 RESULTS

### 3.1 Autofluorescence image change with increasing tumor size

**Figure 3:**
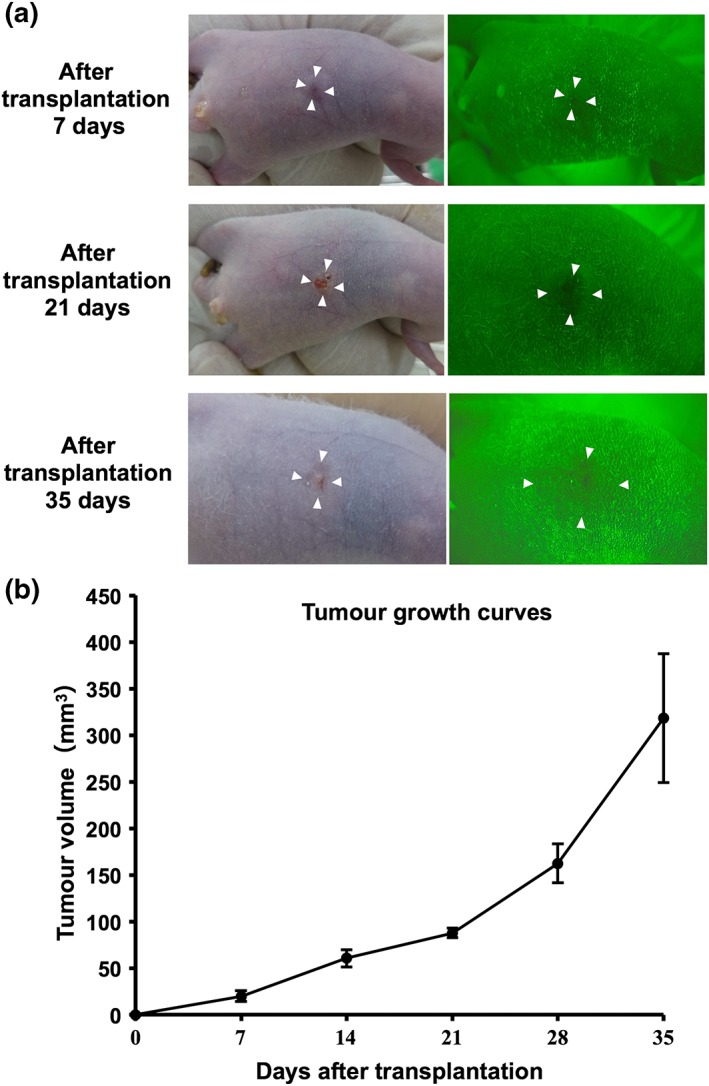
Autofluorescence images of tumor growth over time. (a) White‐light images are presented on the left and autofluorescence images on the right. HSC‐3 human oral squamous cell carcinoma cells were transplanted into the flank of nude mice and observed over time. A tumor is observed as fluorescence visual loss (right). (b) Tumor growth curve for the HSC‐3 xenografts. Mice were inoculated by subcutaneous injection of 2 × 10^6^ HSC‐3 cells on Day 0. Tumor volume was measured every 7 days after transplantation. The error bars represent standard deviation

Tumor engraftment was confirmed 7 days after transplantation of the HSC‐3 human OSCC cell line into the nude mice. Autofluorescence imaging revealed FVL, with the focus of this being the tumor parenchyma (Figure 3a). First, we observed a change in the autofluorescence images as the xenograft mouse model tumor grew, with the tumor parenchyma darkening in the autofluorescence images, the surrounding tissue appearing darker still, and the normal tissue appearing green (Figure 3a).

The tumors were measured every week after tumor implantation and gradually increased in size with an outward bulge. A tumor growth curve was created from the measured volumes, and this showed that mean tumor volume sharply increased from 87.7 mm^3^ at 21 days after implantation to 162.2 mm^3^ at 28 days (Figure 3b).

### 3.2 Luminance change in tumor parenchyma and surrounding tissue and its association with tumor growth

**Figure 4:**
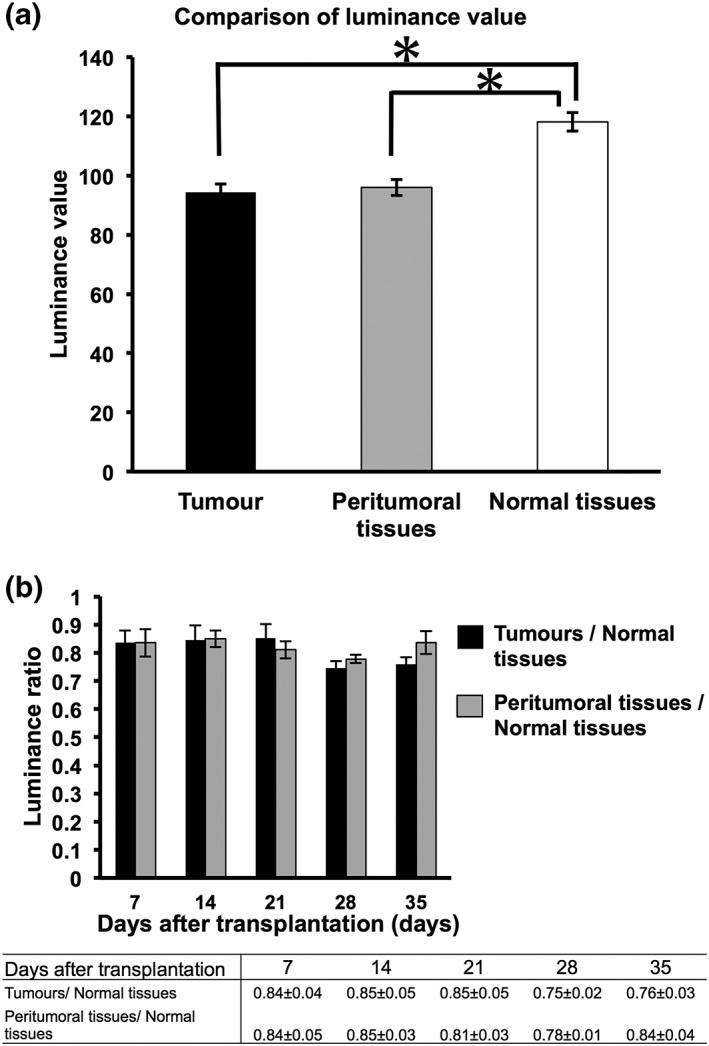
Comparison of the luminance values of the different tissues. (a) Mean values of the luminance of different tissues from all data, including measurements on different days. Error bars represent standard error of the mean (SEM). *p < 0.05. (b) Comparison of the luminance ratios of tumor and peritumoral tissue to normal tissue against number of days posttransplantation. A change in the luminance ratio occurs according to the number of days posttransplantation. All quantitative data are represented as mean ± SEM

The mean luminance values of tumor, surrounding tissue, and normal tissue were 94.3, 96.0, and 118.1 respectively. The luminance values of tumor parenchyma and surrounding tissue were significantly reduced compared with the luminance of normal tissue (Figure 4a). However, there was no significant difference between the luminance of tumor parenchyma and its surrounding tissue (Figure 4a).

To eliminate fluctuations in the luminance values of each image, we observed changes in tumor luminance as the ratio of the luminance of the tumor to that of normal tissue, with the luminance of the normal tissue normalized to a value of 1.0. We hypothesized that the luminance ratio would reduce in association with tumor growth; however, contrary to our expectation, there were no significant differences in luminance ratios in association with tumor growth (Figure 4b). The highest luminance ratio of the tumor parenchyma was 0.85 ± 0.05 at 21 days after tumor transplantation, whereas the highest luminance ratio of the surrounding tissue was 0.84 ± 0.05 at 7 days after tumor transplantation (Figure 4b).

### 3.3 FAD and NADH levels in the tumor tissue of the OSCC human‐xenograft mouse model

**Figure 5:**
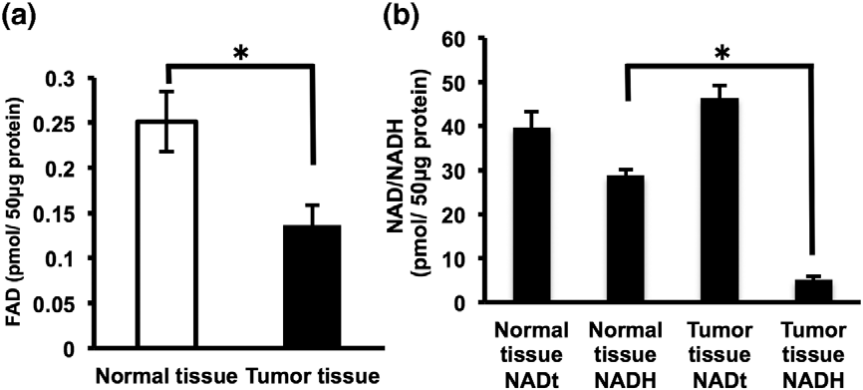
Comparison of the FAD and NADH concentrations in tumors and normal tissues. (a) The FAD values are the mean ± *SD* of two independent experiments. *p < 0.05. (b) NAD or NADH values are the mean ± *SD*. *p < 0.05. NADt includes NAD^+^ and NADH. FAD: flavin adenine dinucleotide; NADH: nicotinamide adenine dinucleotide; SD: standard deviation

The levels of FAD and NADH in a fixed quantity of protein (50 μg) were measured to compare the FAD and NADH levels between the human‐tumor xenograft tissue and normal mouse skin tissue in the OSCC xenograft mouse model. The concentrations of FAD and NADH in normal mouse skin tissue were 0.25 and 29 pmol, respectively, whereas in the human‐tissue xenograft, they were both significantly lower, at 0.14 and 5 pmol, respectively (Figure 5a,b).

## 4 DISCUSSION

The aim of this research was to investigate the value of the autofluorescence luminance ratio of an autofluorescence imaging system for assisting in the identification of OSCC in dental practice and to identify the principal factors affecting changes in this luminance ratio.

In this research, we transplanted the HSC‐3 OSCC cell line into nude mice and observed tumor growth over time, examining the levels of FAD and NADH, which are thought to affect the FVL change in the autofluorescence image. A feature of the luminance ratio is that it decreases when the FVL darkens in comparison with the luminance of normal tissue (Ohnishi et al., 2016; Westra & Sidransky, 2006; Yamamoto et al., 2017). Although there have been a few observations of change in the luminance ratio associated with tumor growth, our results indicated no significant temporal differences in the luminance ratios of tumor parenchyma and surrounding tissue over time (Figure 4b). These findings lead us to suggest that the luminance ratio is stable, irrespective of the stage of tumor growth; when the luminance ratio of a lesion is under 0.90, it can be suspected of being a tumor, as the highest tumor parenchyma luminance ratio measured in all of our data was 0.85 ± 0.05 (Figure 4b). In addition, FAD and NADH, which are considered to be the main factors affecting FVL, were markedly decreased in tumor tissue in comparison with normal tissue (Figure 5).

Over the past three decades, many researchers have reported on the use of autofluorescence visualization devices such as the VELscope for uses including not only screening for OSCC but other clinical applications such as the setting of margins during surgery for other types of cancer (Awan et al., 2011; Elvers et al., 2015; Lane et al., 2006; Onoyama et al., 2016; Poh et al., 2006; Poh et al., 2016; Scheer et al., 2016; Westra & Sidransky, 2006). In general, a reduction in FAD and NADH levels is considered to be a factor influencing the FVL image, with the reason being that cancer cells are often found to undergo a metabolic switch from favoring energy production through oxidative phosphorylation to energy production through aerobic glycolysis (the Warburg effect; Warburg, 1956). Furthermore, coenzymes NADH and FAD are known to be involved in the catabolic reactions of amino acid and fatty acid oxidation, glycolysis, citric acid, and the electron transport chain, which ultimately results in energy generation (Pelicano et al., 2006; Warburg, 1956). Studies using new equipment such as fluorescence lifetime imaging microscopy have shown that the expression of FAD and NADH in cancer cells is decreased in comparison with normal cells (Cannon, Shah, & Skala, 2017; Huang et al., 2017; Scheer et al., 2016; Wallrabe et al., 2018; Yamamoto et al., 2017). Therefore, a reduction in FAD and NADH levels is considered to a factor creating the FVL in autofluorescence visualization images of tumors. However, not many studies have performed a detailed examination of the factors influencing the FVL (Laronde et al., 2014; Luo et al., 2016; Messadi, 2013; Schantz et al., 1998). In this study, we compared the levels of FAD and NADH between tumor tissue and normal skin tissue and revealed that the FAD and NADH levels of tumor tissue were significantly lower than normal tissue (Figure 5a,b). Furthermore, observation with the autofluorescence visualization device after tumor transplantation revealed no change in the luminance ratio after tumor growth and expansion, thereby indicating that the luminance ratio is stable and is not affected by tumor growth (Figure 4).

In this study, we used the Illumiscan device for autofluorescence visualization; the developers state that this device is specialized for the detection of fluorescence from FAD and that it is equipped with a fluorescence filter that interrupts wavelengths shorter than 470 nm. However, in the tumor tissue, not only was the amount of FAD lower than in normal tissue but also the amount of NADH; therefore, a cutoff value may also be helpful with other devices such as the TEL scope, although this would of course need to be examined in detail.

This study used an OSCC xenograft mouse model to demonstrate a cutoff value for the luminance ratio that would be valid over time. This model involved human squamous cell carcinoma cells being inoculated under the hypodermal tissue of the mice. In the autofluorescence observations made during the tumor growth, normal mouse skin epidermal cells were present above the cancer tissue, and the autofluorescence signal may have therefore been partly masked in this mouse model. This means that the luminance ratio will require further definition, and in clinical practice, it may be lower than the value determined here. Although a cutoff value cannot be put it into clinical practice on its own, the method of determining a luminance ratio with normal tissue set to 1.0 should be useful, and we suggest that a cutoff value of 0.90 could be used as a starting criterion. Above all, this study clearly indicates that tumor growth and/or expansion does not affect the luminance ratio in this mouse model, as we observed the luminance ratios to be stable over periods of tumor growth.

To conclude, we demonstrated that the autofluorescence luminance values of tumor and its surrounding tissue were clearly lower than those of normal tissue in a mouse xenograft model and that when the luminance of normal tissue is set to 1.0, a luminance ratio under 0.90 could be useful for suggesting the presence of OSCC in this mouse model.

## CONFLICT OF INTEREST

None declared.

## References

[cre2126-bib-0001] Awan, K. H. , Morgan, P. R. , & Warnakulasuriya, S. (2011). Evaluation of an autofluorescence based imaging system (VELscope) in the detection of oral potentially malignant disorders and benign keratoses. Oral Oncology, 47, 274–277. 21396880 10.1016/j.oraloncology.2011.02.001

[cre2126-bib-0002] Bin‐Shuwaish, M. , Yaman, P. , Dennison, J. , & Neiva, G. (2008). The correlation of DIFOTI to clinical and radiographic images in Class II carious lesions. Journal of the American Dental 323 Association (1939), 139, 1374–1381. 10.14219/jada.archive.2008.0049 18832273

[cre2126-bib-0003] Cannon, T. M. , Shah, A. T. , & Skala, M. C. (2017). Autofluorescence imaging captures heterogeneous drug response differences between 2D and 3D breast cancer cultures. Biomedical Optics Express, 8, 1911–1925. 28663873 10.1364/BOE.8.001911 PMC5480588

[cre2126-bib-0004] Divisi, D. , Di Tommaso, S. , De Vico, A. , & Crisci, R. (2010). Early diagnosis of lung cancer using a SAFE‐3000 autofluorescence bronchoscopy. Interactive Cardiovascular and Thoracic Surgery, 11, 740–744. 20852332 10.1510/icvts.2010.242123

[cre2126-bib-0005] Elvers, D. , Braunschweig, T. , Hilgers, R. D. , Ghassemi, A. , Mohlhenrich, S. C. , Holzle, F. , … Modabber, A. (2015). Margins of oral leukoplakia: Autofluorescence and histopathology. The British Journal of Oral & Maxillofacial Surgery, 53, 164–169. 25434724 10.1016/j.bjoms.2014.11.004

[cre2126-bib-0006] Farah, C. S. , McIntosh, L. , Georgiou, A. , & McCullough, M. J. (2012). Efficacy of tissue autofluorescence imaging (VELScope) in the visualization of oral mucosal lesions. Head & neck, 34, 856–862. 21818819 10.1002/hed.21834

[cre2126-bib-0007] Ferreri, A. J. , Illerhaus, G. , Zucca, E. , Cavalli, F. , & International Extranodal Lymphoma Study G (2010). Flows and flaws in primary central nervous system lymphoma. Nature reviews. Clinical oncology, 7: doi: 10.1038/nrclinonc.2010%209-c1; author reply, 1–2.doi: 10.1038/nrclinonc.2010 9‐c2 20700952

[cre2126-bib-0008] Ganga, R. S. , Gundre, D. , Bansal, S. , Shirsat, P. M. , Prasad, P. , & Desai, R. S. (2017). Evaluation of the diagnostic efficacy and spectrum of autofluorescence of benign, dysplastic and malignant lesions of the oral cavity using VELscope. Oral Oncology, 75, 67–74. 29224826 10.1016/j.oraloncology.2017.10.023

[cre2126-bib-0009] Hanken, H. , Kraatz, J. , Smeets, R. , Heiland, M. , Assaf, A. T. , Blessmann, M. , … Rana, M. (2013). The detection of oral pre‐malignant lesions with an autofluorescence based imaging system (VELscope)—A single blinded clinical evaluation. Head & Face Medicine, 9, 23. 23967796 10.1186/1746-160X-9-23 PMC3851797

[cre2126-bib-0010] Huang, T. T. , Huang, J. S. , Wang, Y. Y. , Chen, K. C. , Wong, T. Y. , Chen, Y. C. , … Chung, P. C. (2017). Novel quantitative analysis of autofluorescence images for oral cancer screening. Oral Oncology, 68, 20–26. 28438288 10.1016/j.oraloncology.2017.03.003

[cre2126-bib-0011] Kato, M. , Kaise, M. , Yonezawa, J. , Yoshida, Y. , & Tajiri, H. (2007). Autofluorescence endoscopy versus conventional white light endoscopy for the detection of superficial gastric neoplasia: A prospective comparative study. Endoscopy, 39, 937–941. 18008201 10.1055/s-2007-966857

[cre2126-bib-0012] Lane, P. M. , Gilhuly, T. , Whitehead, P. , Zeng, H. , Poh, C. F. , Ng, S. , … MacAulay, C. E. (2006). Simple device for the direct visualization of oral‐cavity tissue fluorescence. Journal of Biomedical Optics, 11, 024006. 16674196 10.1117/1.2193157

[cre2126-bib-0013] Laronde, D. M. , Williams, P. M. , Hislop, T. G. , Poh, C. , Ng, S. , Bajdik, C. , … Rosin, M. P. (2014). Influence of fluorescence on screening decisions for oral mucosal lesions in community dental practices. Journal of Oral Pathology & Medicine: Official Publication of the International Association of Oral Pathologists and the American Academy of Oral Pathology, 43, 7–13. 10.1111/jop.12090 PMC3835795 23750637

[cre2126-bib-0014] Lingen, M. W. , Kalmar, J. R. , Karrison, T. , & Speight, P. M. (2008). Critical evaluation of diagnostic aids for the detection of oral cancer. Oral Oncology, 44, 10–22. 17825602 10.1016/j.oraloncology.2007.06.011 PMC2424250

[cre2126-bib-0015] Luo, X. , Xu, H. , He, M. , Han, Q. , Wang, H. , Sun, C. , … Chen, Q. (2016). Accuracy of autofluorescence in diagnosing oral squamous cell carcinoma and oral potentially malignant disorders: a comparative study with aero‐digestive lesions. Scientific Reports, 6, 29943. 27416981 10.1038/srep29943 PMC4945954

[cre2126-bib-0016] Mayinger, B. , Horner, P. , Jordan, M. , Gerlach, C. , Horbach, T. , Hohenberger, W. , & Hahn, E. G. (2001). Light‐induced autofluorescence spectroscopy for the endoscopic detection of esophageal cancer. Gastrointestinal Endoscopy, 54, 195–201. 11474390 10.1067/mge.2001.116563

[cre2126-bib-0017] Messadi, D. V. (2013). Diagnostic aids for detection of oral precancerous conditions. International Journal of Oral Science, 5, 59–65. 23743617 10.1038/ijos.2013.24 PMC3707069

[cre2126-bib-0018] Mortazavi, H. , Baharvand, M. , & Mehdipour, M. (2014). Oral potentially malignant disorders: An overview of more than 20 entities. Journal of Dental Research, Dental Clinics, Dental Prospects, 8, 6–14. 10.5681/joddd.2014.002 PMC4091702 25024833

[cre2126-bib-0019] Muir, C. , & Weiland, L. (1995). Upper aerodigestive tract cancers. Cancer, 75, 147–153. 8000993 10.1002/1097-0142(19950101)75:1+<147::aid-cncr2820751304>3.0.co;2-u

[cre2126-bib-0020] Ohnishi, Y. , Fujii, T. , Ugaki, Y. , Yasui, H. , Watanabe, M. , Dateoka, S. , & Kakudo, K. (2016). Usefulness of a fluorescence visualization system for the detection of oral precancerous and early cancerous lesions. Oncology Reports, 36, 514–520. 27121913 10.3892/or.2016.4776

[cre2126-bib-0021] Onoyama, H. , Kamiya, M. , Kuriki, Y. , Komatsu, T. , Abe, H. , Tsuji, Y. , … Seto, Y. (2016). Rapid and sensitive detection of early esophageal squamous cell carcinoma with fluorescence probe targeting dipeptidylpeptidase IV. Scientific Reports, 6, 26399. 27245876 10.1038/srep26399 PMC4887889

[cre2126-bib-0022] Osman, T. A. , Costea, D. E. , & Johannessen, A. C. (2012). The use of salivary cytokines as a screening tool for oral squamous cell carcinoma: A review of the literature. Journal of Oral and Maxillofacial Pathology: JOMFP, 16, 256–261. 22923900 10.4103/0973-029X.99083 PMC3424944

[cre2126-bib-0023] Pelicano, H. , Xu, R. H. , Du, M. , Feng, L. , Sasaki, R. , Carew, J. S. , … Huang, P. (2006). Mitochondrial respiration defects in cancer cells cause activation of Akt survival pathway through a redox‐mediated mechanism. The Journal of Cell Biology, 175, 913–923. 17158952 10.1083/jcb.200512100 PMC2064701

[cre2126-bib-0024] Pentenero, M. , Marino, R. , Tempia Valenta, G. , Navone, R. , & Gandolfo, S. (2014). Microbiopsy a novel sampling technique to early detect dysplastic/malignant alterations in oral mucosal lesions: Practicability by general dentists. Journal of Oral Pathology & Medicine: Official Publication of the International Association of Oral Pathologists and the American Academy of Oral Pathology, 43, 435–440. 10.1111/jop.12161 24484286

[cre2126-bib-0025] Pentenero, M. , Val, M. , Rosso, S. , & Gandolfo, S. (2018). Microbiopsy a first‐level diagnostic test to rule out oral dysplasia or carcinoma in general dental practice. Oral Diseases, 24, 109–111. 29480597 10.1111/odi.12735

[cre2126-bib-0026] Petersen, P. E. (2009). Oral cancer prevention and control—The approach of the World Health Organization. Oral Oncology, 45, 454–460. 18804412 10.1016/j.oraloncology.2008.05.023

[cre2126-bib-0027] Petruzzi, M. , Lucchese, A. , Nardi, G. M. , Lauritano, D. , Favia, G. , Serpico, R. , & Grassi, F. R. (2014). Evaluation of autofluorescence and toluidine blue in the differentiation of oral dysplastic and neoplastic lesions from non dysplastic and neoplastic lesions: A cross‐sectional study. Journal of Biomedical Optics, 19, 76003. 24996662 10.1117/1.JBO.19.7.076003

[cre2126-bib-0028] Poh, C. F. , Anderson, D. W. , Durham, J. S. , Chen, J. , Berean, K. W. , MacAulay, C. E. , & Rosin, M. P. (2016). Fluorescence visualization‐guided surgery for early‐stage oral cancer. JAMA Otolaryngology. Head & Neck Surgery, 142, 209–216. 26769431 10.1001/jamaoto.2015.3211

[cre2126-bib-0029] Poh, C. F. , Zhang, L. , Anderson, D. W. , Durham, J. S. , Williams, P. M. , Priddy, R. W. , … Rosin, M. P. (2006). Fluorescence visualization detection of field alterations in tumor margins of oral cancer patients. Clinical Cancer Research: An Official Journal of the American Association for Cancer Research, 12, 6716–6722. 17121891 10.1158/1078-0432.CCR-06-1317

[cre2126-bib-0030] Rhodus, N. L. (2009). Oral cancer and precancer: Improving outcomes. Compendium of continuing education in dentistry (Jamesburg, N.J. : 1995), 30, 486–488. 490‐4, 496‐8 passim; quiz 504, 520 19824564

[cre2126-bib-0031] Schantz, S. P. , Kolli, V. , Savage, H. E. , Yu, G. , Shah, J. P. , Harris, D. E. , … Huvos, A. G. (1998). In vivo native cellular fluorescence and histological characteristics of head and neck cancer. Clinical Cancer Research: An Official Journal of the American Association for Cancer Research, 4, 1177–1182. 9607575

[cre2126-bib-0032] Scheer, M. , Fuss, J. , Derman, M. A. , Kreppel, M. , Neugebauer, J. , Rothamel, D. , … Zoeller, J. E. (2016). Autofluorescence imaging in recurrent oral squamous cell carcinoma. Oral and Maxillofacial Surgery, 20, 27–33. 26267490 10.1007/s10006-015-0520-7

[cre2126-bib-0033] Scott, S. E. , Grunfeld, E. A. , & McGurk, M. (2005). The idiosyncratic relationship between diagnostic delay and stage of oral squamous cell carcinoma. Oral Oncology, 41, 396–403. 15792612 10.1016/j.oraloncology.2004.10.010

[cre2126-bib-0034] Shin, D. , Vigneswaran, N. , Gillenwater, A. , & Richards‐Kortum, R. (2010). Advances in fluorescence imaging techniques to detect oral cancer and its precursors. Future Oncology, 6, 1143–1154. 20624126 10.2217/fon.10.79 PMC2929485

[cre2126-bib-0035] Vernham, G. A. , & Crowther, J. A. (1994). Head and neck carcinoma—Stage at presentation. Clinical Otolaryngology and Allied Sciences, 19, 120–124. 8026088 10.1111/j.1365-2273.1994.tb01194.x

[cre2126-bib-0036] Wallrabe, H. , Svindrych, Z. , Alam, S. R. , Siller, K. H. , Wang, T. , Kashatus, D. , … Periasamy, A. (2018). Segmented cell analyses to measure redox states of autofluorescent NAD (P) H, FAD & Trp in cancer cells by FLIM. Scientific Reports, 8, 79. 29311591 10.1038/s41598-017-18634-x PMC5758727

[cre2126-bib-0037] Warburg, O. (1956). On the origin of cancer cells. Science (New York, N.Y.), 123, 309–314. 10.1126/science.123.3191.309 13298683

[cre2126-bib-0038] Westra, W. H. , & Sidransky, D. (2006). Fluorescence visualization in oral neoplasia: Shedding light on an old problem. Clinical Cancer Research: An Official Journal of the American Association for Cancer Research, 12, 6594–6597. 17121876 10.1158/1078-0432.CCR-06-2253

[cre2126-bib-0039] Yamamoto, N. , Kawaguchi, K. , Fujihara, H. , Hasebe, M. , Kishi, Y. , Yasukawa, M. , … Hamada, Y. (2017). Detection accuracy for epithelial dysplasia using an objective autofluorescence visualization method based on the luminance ratio. International Journal of Oral Science, 9, e2. 29125138 10.1038/ijos.2017.37 PMC5775331

